# Evaluation of left ventricular torsion by cardiovascular magnetic resonance

**DOI:** 10.1186/1532-429X-14-49

**Published:** 2012-07-24

**Authors:** Alistair A Young, Brett R Cowan

**Affiliations:** 1 Department of Anatomy with Radiology, University of Auckland, Auckland, New Zealand

## Abstract

Recently there has been considerable interest in LV torsion and its relationship with symptomatic and pre-symptomatic disease processes. Torsion gives useful additional information about myocardial tissue performance in both systolic and diastolic function. CMR assessment of LV torsion is simply and efficiently performed. However, there is currently a wide variation in the reporting of torsional motion and the procedures used for its calculation. For example, torsion has been presented as twist (degrees), twist per length (degrees/mm), shear angle (degrees), and shear strain (dimensionless). This paper reviews current clinical applications and shows how torsion can give insights into LV mechanics and the influence of LV geometry and myocyte fiber architecture on cardiac function. Finally, it provides recommendations for CMR measurement protocols, attempts to stimulate standardization of torsion calculation, and suggests areas of useful future research.

## Review

### Background

**Figure 1:**
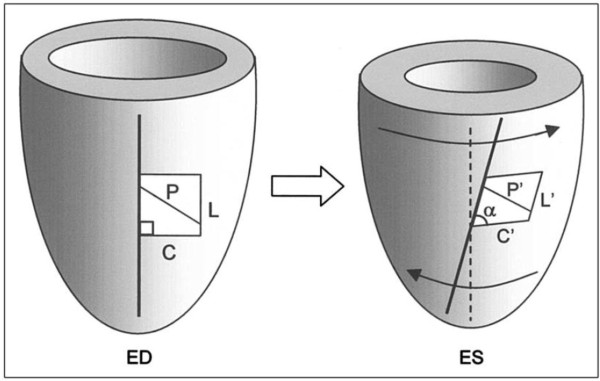
**Axial and shear strains during systole.** Relative to ED (left), the LV at ES (right) has shortened in both the longitudinal (L → L’) and circumferential (C → C’) directions (axial strains). A characteristic wringing motion (arrows) gives rise to a torsional shear given by the angle θ_CL_ = 90°- α. One significant effect of torsion is that the greatest shortening (P → P’) occurs obliquely to C and L, in the approximate direction of the sub-epicardial fibers. From [8] © American Journal of Cardiology, used by permission.

Left ventricular torsion has long been recognized as a characteristic of normal mammalian cardiac function, described by William Harvey and many others [1,2]. Relative to end-diastole (ED) the apex of the left ventricle rotates anticlockwise about its central axis, as viewed from the apex, at a relatively constant rate throughout systole, to a maximum value of ~10° [3,4]. The base, initially rotating anticlockwise, reverses direction to give a net clockwise rotation by end-systole (ES) of ~3° [3,4]. The resulting end-systolic torsion (defined to be positive by convention) is often described as being similar to wringing out a wet towel (see Additional file 1 and Additional file 2, and Figure 1). During diastole much of the systolic torsion is released during isovolumic relaxation, due to the mechanical recoil of elastic energy built up during systole [5-7]. Thus, relaxation of torsion is a direct measure of the deactivation of myocytes and release of stored elastic energy, both of which facilitate rapid filling.

Torsion has recently gained increasing attention due to two factors. Firstly, simple and direct methods of quantification by non-invasive imaging are now widely available. Initially measured using invasively implanted radiopaque or ultrasonic markers [9,10], the current gold standard for evaluation of LV torsion is by CMR tissue tagging [11], but it can also be quantified with echocardiographic speckle tracking [12]. Secondly, torsion is a useful and interesting index of cardiac performance which provides important information on myocardial mechanics that complements standard pump function indices. Torsional deformation is sensitive to changes in endocardial and epicardial contraction, concentric remodeling and the fibrous architecture of the heart. It can therefore provide insight into the mechanical processes of normal and abnormal cardiac function during both contraction and relaxation.

Torsion is remarkably consistent across mammalian species, but is affected (either increased or decreased) by a variety of pathologies, including cardiomyopathies [13], diabetes [8], hypertrophy [14], hypertension [15], and ischemia [16], as well as normal aging [17].

This review firstly provides a discussion of how torsion can provide useful additional information on LV mechanical function, followed by an overview of the current clinical applications of LV torsion. It then provides recommendations for standardized protocols for measuring and reporting torsional motion, and finally suggests some useful avenues for future research.

### Torsion in LV mechanics

#### The force balance

**Figure 2:**
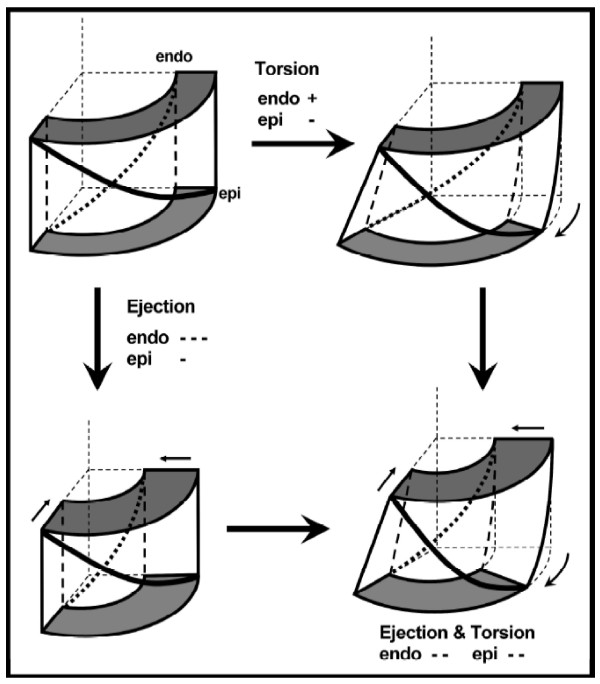
**Torsion equalizes transmural fiber strain.** Subepicardial muscle fibers (solid thick line) shorten but subendocardial fibers (dotted line) lengthen under the action of positive torsion without contraction (top right). Contraction without torsion leads to greater fiber shortening in the subendocardium due to incompressibility (bottom left). Combined contraction and torsion leads to equal fiber shortening on epicardium and endocardium (bottom right). From [17] © American Journal of Physiology, used by permission.

In this paper we define the relative rotation between the apex and base as “twist” and the resulting shear angle (θ_CL_ = 90°- α in Figure 1) as “torsion”. Torsion therefore describes the shear deformation undergone by the myocardium and is preferred to twist since, for the same torsion, the twist can be variable depending on heart length and diameter. Torsion results from the structural architecture of the mammalian heart, in which subepicardial myofibers are oriented approximately 60 degrees below circumferential in a left handed helix, midwall fibers are oriented circumferentially and subendocardial fibers are oriented approximately 60 degrees above circumferential in a right handed helix (Figure 2). This characteristic architecture gives rise to torsional shear strain as well as axial (circumferential and longitudinal) strain (Figure 1). Mechanical models of ventricular contraction can give insight into the balance of mechanical forces between the different fiber orientations, which leads to the characteristic torsion pattern seen in vivo [18,19]. Contraction of the left handed epicardial fibers adds to positive (left handed) systolic torsion while contraction of subendocardial fibers opposes positive torsion. The force balance is typically in favor of the epicardial fibers due to their increased lever arm, given myofiber shortening is relatively uniform across the wall. Torsion therefore gives information about the mechanical function of different fiber populations. The presence of torsion facilitates homogeneous fiber shortening across the wall (Figure 2). In the absence of torsion, endocardial fiber shortening would be greater than seen *in vivo* (Figure 2), due to the mechanical effects of the contraction of the nearly incompressible myocardium. Torsion also gives rise to a principal shortening direction (i.e. direction of maximum contraction, P in Figure 1) which is oriented obliquely to the short axis plane, in the approximate direction of the subepicardial muscle fibers. The direction of the principal shortening is relatively constant across the wall, despite the large change in fiber angle, so that the maximum contraction in the subendocardial wall is approximately orthogonal to the fiber direction [20].

The force balance can be altered by changes in the geometry of the heart. For example LV concentric hypertrophy typically leads to increased torsion due to the thicker heart wall. Epicardial myofibers have a proportionally greater lever arm than endocardial fibers and thus contribute to greater torsion. Conversely eccentric hypertrophy typically leads to decreased torsion.

#### Torsion and myocardial architecture

Mathematical models have shown that the normal distribution of myofiber orientation in healthy subjects gives rise to a particular value of torsion which equilibrates fiber contraction and fiber stress across the wall, so that myocytes experience the same force from epicardium to endocardium [18,21]. Torsion is markedly different in conditions where the myofiber architecture is not normal. For example, in situs inversus totalis the apical and epicardial basal fiber orientation is normal but the deeper basal fibers have an inverted fiber orientation [22]. Torsion is consequently normal at the apex but changes sign towards the base [23-25]. There is typically no relative rotation between the apex and base, but the midventricular region rotates clockwise relative to both [24] viewed from the apex. Mathematical modeling shows that the predicted torsion arising from a force balance is consistent with that observed *in vivo*[25].

The remarkable uniformity of fiber strain compared with circumferential or radial strain has led some authors to hypothesize that fiber architecture may adapt in response to strain or force signals to optimize the uniformity of fiber contraction [21]. Simple adaptation laws minimizing cross-fiber shear can act to homogenize fiber strain [26]. The extent to which fiber architecture can adapt under the influence of mechanical signals is an interesting area of future study.

#### Descriptions of myocardial architecture

In addition to the myofiber helix angle described above, myofibers can have an imbrication or transverse angle, particularly near the apex and base, in which myofibers travel across the wall from epicardium to endocardium or vice versa [27]. Myocardium is also organized into laminae about 4 cells thick, whose orientations vary in a complex pattern [28]. These laminae are thought to facilitate wall thickening during systole [28]. Due to the complex nature of myocardial architecture, several attempts have been made to rationalize this structure according to geodesics [29], or bands [30]. These can be thought of as conceptual interpretations of the three-dimensional branching syncytium [31], but are not recommended for the interpretation of mechanical function. The continuum force balance approach described above has been more useful for this purpose [32].

#### Torsion to shortening ratio

Although torsion itself is load dependent [33], the torsion-volume relation is relatively load independent [34]. Mathematical models have shown that a specific relationship between torsion and ejection is required to balance forces and maintain a uniform fiber shortening across the wall [35,36]. This leads to a consistent ratio between shortening and torsion during systole across mammalian species, known as the torsion-to-shortening ratio (TSR) [37], which is theoretically independent of contractility, afterload and preload. Changes in this ratio therefore indicate transmural differences in fiber contraction. In particular, models predict that impaired subendocardial function leads to increased TSR, due to a reduced opposition to positive torsion (Figure 2). TSR has been found to be substantially increased in patients with aortic stenosis [37], who are known to have impaired subendocardial function. Smaller increases in TSR can also be seen in normal aging [17], consistent with the hypothesis that endocardial function reduces with age. Russel *et al.*[38] found increased torsion and TSR in HCM mutation carriers with normal wall thickness, perhaps indicating preclinical subendocardial dysfunction.

#### Activation and deactivation

The entire LV is observed to initially rotate counter-clockwise. This may be due to the anterior epicardial myofibers extending across the interventricular sulcus to the RV and great vessels [39]. Alternatively, the initial counter-clockwise rotation might be due to the orientation of the great vessels, since it is reversed in situs inversus totalis [24].

During diastole, the recoil rate (rate of relaxation of torsion to end-diastolic values), is faster than the relaxation of axial strains in healthy volunteers [40]. Although torsion typically increases approximately linearly with ejection (relative to end-diastole), much of the end-systolic torsion is typically released during iso-volumic relaxation [5,6]. This is likely due to the mechanical release of stored elastic energy in the myocardial tissue [5] and is correlated with the rapid pressure fall and fast inflow during rapid filling [7]. The recoil rate has been shown to be correlated with the time constant of pressure decay, and intraventricular pressure gradients, during relaxation in dogs [41,42] with volume and inotropic interventions. However, some studies in humans with diastolic dysfunction did not show decreased recoil rate, rather the main determinants of recoil rate were peak twist and end systolic volume [43,44].

Another factor influencing the generation of peak torsion and recoil rate may be the phosphorylation of myosin light chain regulatory proteins [45,46]. There is a spatial gradient of myosin light chain phosphorylation across the heart wall (from higher values in the epicardium to lower values in the endocardium) [45]. This increases left handed torsion since myosin light chain phosphorylation is associated with increased isometric tension and decreased stretch activation response [45]. In double knockout mice mutants with reduced myosin light chain phosphorylation, reduced torsion and recoil rate were observed consistent with reduced epicardial tension, which were confirmed by multiscale modeling of myosin cycling kinetics in a mechanical model [46].

#### Torsion and transverse shear

**Figure 3:**
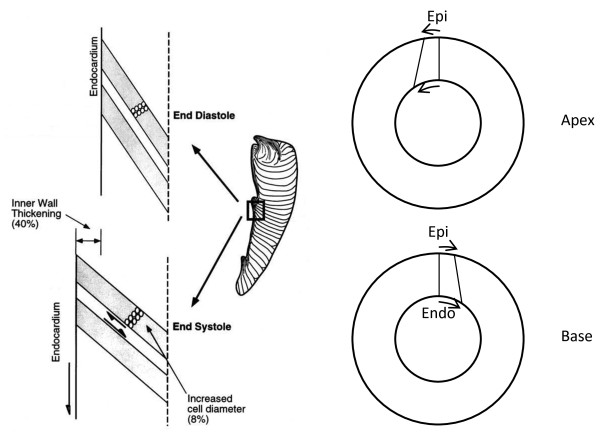
**Transverse shears.** Left: longitudinal-radial transverse shear is facilitated by myocardial laminae (modified from [47]). Right: circumferential-radial transverse shear is facilitated by increased torsion on the endocardium relative to the epicardium.

Although torsion is a shear deformation and therefore volume preserving, it may contribute indirectly to the ejection of blood and the thickening of the ventricular wall during systole. Several studies have noted that *transverse shears*, in which cells slide over one another in the radial (transmural) direction, have a major contribution to the substantial wall thickening observed during systole [47] (Figure 3). *Longitudinal-radial* transverse shears have been shown to be mechanically facilitated by myocardial laminae, and maximum local shearing is aligned with the laminae orientation in the subendocardium [47-49]. Another type of transverse shear arises due to a difference in systolic rotational motion between epicardium and endocardium (*circumferential-radial shear*). CMR tagging studies have shown that the apical anticlockwise rotation is greater at the endocardium than the epicardium, and the basal clockwise rotation is greater at the endocardium than the epicardium [4,10,14]. This characteristic variation in circumferential-radial shear gives rise to an increase in torsion towards the endocardium [14,40]. This is mechanically paradoxical since subendocardial fibers should act to reduce torsion towards the subendocardium, not increase it. One possible mechanism may be the transverse angle of myocardial fibers near the apex and base which can more effectively transmit epicardial forces to the endocardium [50,51]. Although there is some evidence of transverse angle from diffusion tensor MRI [27], whether this is sufficient to predict the observed transverse shear is not yet known. The transmural variation of torsion therefore clearly requires further study.

### Clinical applications

#### Aortic stenosis

Patients with pressure overload hypertrophy due to aortic stenosis typically display increased torsion and prolonged torsional recoil relative to healthy controls or athletes with physiological volume-overload hypertrophy [15,52]. This may be indicative of reduced subendocardial function due to regional ischemia, thereby impairing the usual action of subendocardial fibers to oppose torsion [53]. After aortic valve replacement, torsion can be somewhat normalized although still elevated [54].

#### Ischemia

In acute ischemia, apical rotation may be initially increased due to relative impairment of subendocardial fibers [55]. In regional ischemia or myocardial infarction, torsion is typically impaired in relation to the regional nature of the disease [11]. In patients with anterolateral myocardial infarction, twist is reduced and untwisting is delayed and prolonged [56-58]. In patients with first time ST elevation myocardial infarction, subendocardial measures of twist appear to be affected before subepicardial twist [16].

#### Hypertrophy

Torsion is known to be dependent on LV shape, with reduced twist in more spherical shaped hearts [59] and increased torsion with concentric hypertrophy due to an increased lever arm for epicardial fibers. In hypertrophic cardiomyopathy torsion was found to be increased despite reduced circumferential and longitudinal shortening [14]. Torsion was also increased despite reduced axial strains in patients with type 2 diabetes with diastolic dysfunction but normal ejection fraction [8]. In mild hypertrophy associated with successful repair of coarctation of the aorta [60], torsion was increased despite reduced longitudinal shortening and maintained circumferential shortening. Part of this effect may be due to reduced subendocardial shortening. For example, torsion and TSR were also found to be increased in healthy carriers of familial HCM with normal wall thickness [38], perhaps indicating preclinical subendocardial disease.

In volume overload hypertrophy, torsion is typically decreased. Torsion and TSR were reduced after experimentally induced mitral regurgitation in animal studies [61,62]. Animals with mitral regurgitation due to infarction showed significantly less torsion and recoil than animals with infarction but no mitral regurgitation [63]. In humans chronic mitral regurgitation leads to reduced torsion and recoil rate [64].

In the physiological hypertrophy seen in elite athletes, torsion can be normal despite increased volume and mass [52]. In a study of athletes imaged before and after running a marathon, both torsion and the magnitude of diastolic recoil were increased, although axial strains were not changed [65]. This finding may be consistent with mild ischemia in the subendocardial layers, but there was no evidence of late gadolinium enhancement [65].

#### Dyssynchrony

In a study of cardiac resynchronization therapy (CRT) in patients with LV dyssynchrony due to heart failure, LV twist and torsion were negatively correlated with radial dyssynchrony (difference between earliest and latest segmental peak radial motion), and these were significantly improved after CRT [66]. Twist and torsion were also the best predictors of CRT responders [66]. In contrast, another study found no improvement in twist after CRT [67]. The difference may be due to lead placement, since LV leads positioned in midventricular and apical regions can exhibit a larger increase in systolic LV twist than LV leads positioned in the basal regions of the LV free wall [68]. This finding may be due to the propagation of the activation wavefront, in particular the transmural activation pattern [69]. Sorger *et al.* have also shown the effects of ectopic activation on torsion development [70].

#### Diastolic dysfunction

In pressure overload due to aortic stenosis, relaxation is delayed [15]. The apical untwist is also delayed in older asymptomatic volunteers [71]. In patients with heart failure with preserved ejection fraction, peak twist can be greater than normal in patients with abnormal relaxation (grade 1) decreasing with pseudonormalization pattern (grade 2) and restrictive pattern (grade 3) [72].

#### Diabetes

In patients with type 2 diabetes with normal ejection fraction but echocardiographic evidence of diastolic dysfunction, peak systolic torsion was increased relative to controls although peak recoil rate was unchanged [8]. Whether this is due to an increased lever arm due to hypertrophy or a reduced subendocardial fiber shortening is currently unclear. Also, in asymptomatic Type I diabetes patients without morphological evidence of cardiac disease [73], LV torsion was increased despite unaltered circumferential strain, consistent with subendocardial dysfunction due to small vessel disease.

#### Cardiac iron overload

In patients with significant iron overload due to repeated transfusions with normal ejection fraction and without heart failure, LV twist was reduced prior to changes in pump function [74]. Torsion and torsional recoil rate were also found to be reduced in patients with transfusion-induced haemochromatosis with myocardial T2* < 10 msec [75].

#### Heart failure

Tachycardia induced heart failure was associated with decreased and delayed systolic torsion and loss of early diastolic recoil [76]. Dilated cardiomyopathy can lead to substantially decreased torsion and earlier peak torsion [13].

#### Evaluation of interventions

Hansen *et al *[9] reported a significant relationship between reductions in torsion and episode of transplant rejection. In a recent study of transplant recipients, a reduction of 25 % or more in torsion predicted Grade 2 or higher rejection with a predictive accuracy of 93 % [77]. Transplant recipients also show less torsion augmentation on exercise than donor-age matched volunteers [78].

In patients with ischemic dilated cardiomyopathy who underwent ventricular reconstruction surgery, those patients with the most reduced torsion before reconstruction showed increased torsion afterwards, but over all patients torsion was not significantly increased [79]. However, torsional recoil rates were increased after ventricular reconstruction surgery over all patients.

#### Transgenic animal studies

Torsion may also be a useful biomarker in the study of mechanical effects of genetically manipulated animal models of cardiac disease. In a mouse model of Duchenne muscular dystrophy Li *et al.*[80] showed initial increased torsion and axial strains in early stages with no fibrosis, and decreased torsion and strain correlating with increased fibrosis in later stages of disease. Torsional changes have also been used to investigate mechanical dysfunction in genetically engineered mouse models of cardiovascular disease [81,82].

### Recommendations

A variety of CMR imaging protocols can be used to evaluate LV torsion, but currently the relative benefits of each are not known. Torsion can be quantified using velocity encoded tissue phase mapping [39], spatial modulation of magnetization (SPAMM) [14], complementary spatial modulation of magnetization [52], harmonic phase analysis [81]. Fast methods of analysis have been proposed using the k-space harmonic peaks [83]. Recently, Nasiraei Moghaddam *et al.* describe a method whereby torsion can be calculated from a single long axis slice using displacement encoded stimulated echo (DENSE) imaging [84]. It may also be possible to quantify torsion from standard SSFP untagged images, using image feature tracking methods [85]. The most complete information however is likely to be provided by 3D displacement encoded CMR imaging, such as 3D DENSE [86].

An example protocol using standard SPAMM imaging would include short axis cine gradient recalled echo segmented k-space SPAMM tagged acquisition with a flip angle of 5-10° degrees (smaller for better contrast in diastole) grid tagging 45° to readout direction with 7 mm spacing, slice thickness 6 mm, repetition time 8 ms, echo time 4 ms, 9 segment view sharing, bandwidth 200 Hz/pixel, giving 23 frames for a 13 sec breath-hold duration. Slices should include at least two short axis locations, one near the apex but including LV cavity at end-systole, and another near the base but including a full circumference of myocardium at end-systole. Reported reproducibility for twist measurements are typically 0.1° for interobserver reproducibility [3].

There is currently a lack of standardization for methods used to characterize the twisting motion of the left ventricle. For example, torsion has been calculated as relative rotation (degrees) [11,65], rotation per length (degrees/mm) [52], torsional shear angle (degrees) [36], and shear strain (dimensionless) [14]. A simple difference in rotation between apex and base (often called *twist*) is not recommended, since this depends on the exact locations of the slices and is difficult to reproduce in longitudinal studies. Twist per unit length of the ventricle is more robust to slice position, since torsion is relatively constant in the longitudinal direction [87], but this measure does not scale appropriately between hearts of different sizes (e.g. mice and humans have comparable ventricular torsion but quite different twist per length). The *torsional shear angle* shown in Figure 1 is a measure of the change in angle between line segments which are initially aligned with the anatomical circumferential and longitudinal axes of the LV. This measure is independent of size and can be calculated at any point in the ventricle. However, there are several ways in which this can be calculated, as briefly outlined below.

In solid mechanics, the 3D strain state at any point in a body can be fully represented by three axial strains and three shear strains [88]. Referred to the anatomical circumferential, longitudinal and transmural coordinates of the left ventricle, the 3D torsional shear angle is given by

(1) $$\sin\theta_{\mathrm{CL}}=\frac{2E_{\mathrm{CL}}}{\sqrt{1+2E_{\mathrm{CC}}}\sqrt{1+2E_{\mathrm{LL}}}}$$

where *E*_*CC*_ is the circumferential axial strain, *E*_*LL*_ is the longitudinal axial strain, and *E*_*CL*_ is the circumferential-longitudinal shear. Russel *et al.*[87] have noted that the 3D torsional shear angle calculated from the 3D strain tensor (Figure 1) can be influenced by circumferential variation in longitudinal displacement, as well as torsion. Since longitudinal displacement is relatively uniform in the circumferential direction this is typically not a significant effect, and averaging around the circumference will eliminate this variation.

**Figure 4:**
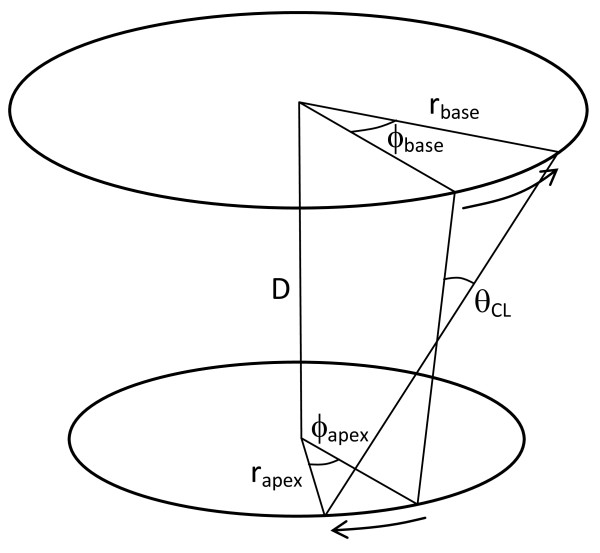
**Calculation of torsional shear angle from two short axis slices, one basal and one apical.***r*_*apex*_*, r*_*base*_: average radius of the cross-section at apical and basal slices respectively. ϕ_*apex*_, ϕ_*base*_: average rotation of the cross section at apical and basal slices respectively. D: distance between slices. *θ*_*CL*_: torsional shear angle. Modified from [89].

A two-dimensional approximation of the torsional shear angle can be calculated from the relative rotation of two short axis slices, one basal and one apical in location (Figure 4).

Many studies have used the formula given by Aelen *et al.*[36] which approximates the torsional shear angle by:

(2) $$\theta_{\mathrm{CL}}=\frac{\left(\varphi_{\mathrm{apex}}-\varphi_{\mathrm{base}})(r_{\mathrm{apex}}+r_{\mathrm{base}}\right)}{2D}$$

where D is the distance between slices. Intuitively, this approximates the shear angle from the relative circumferential displacement of the base and apex (rϕ), assuming tan(θ)≈θ for small angles. However, this formula assumes that the radii of the apex and base are approximately the same, and can overestimate torsional shear relative to a 3D method [89]. An alternative formula given by Russel *et al. *[89] calculates the difference in circumferential displacement directly:

(3) $$\theta_{\mathrm{CL}}=\frac{\left(\varphi_{\mathrm{apex}}r_{\mathrm{apex}}-\varphi_{\mathrm{base}}r_{\mathrm{base}}\right)}{D}$$

This version has been found to give unbiased estimates of the 3D torsional shear angle even in cases with high torsion [89].

Since it has been used in many previous studies, is easy to understand, and relatively invariant to ventricular size and slice position, equation 2 is recommended for the simple calculation of torsion from two short axis slices. However, for direct comparison with the 3D torsional shear, equation 3 is more appropriate. Regional torsion, for example referred to the standard 17 segment model, should not be calculated using either equations 2 or 3, since regional estimates are highly sensitive to the exact position of the axis of rotation [87]. For regional estimates, the 3D torsional shear (equation 1) is recommended.

For diastolic function, increased torsion generally leads to increased torsional recoil [90,91]. For example, peak torsion was increased in patients with type 2 diabetes, diastolic dysfunction and normal ejection fraction, whereas torsional recoil rate was normal [8], leading to impaired recoil relative to the peak torsion. Therefore torsional recoil rate should be normalized by peak systolic torsion.

### Future work

Investigation of the relationship between torsional deformation and the microstructure of the heart, together with the changes due to disease, is likely to be a fruitful area of future research. Detailed information on the myofiber architecture can be obtained from diffusion tensor and diffusion spectrum imaging [92,93]. Although to date most studies have required isolated arrested hearts, in-vivo measurement of myofiber architecture is an active area of current research [94].

One interesting open question is whether patients with heart failure and normal ejection fraction have impaired relaxation of torsion, since some studies have not found any significant difference [44]. One hypothesis is that, like diabetic patients with normal ejection fraction and grade 1 diastolic dysfunction [8], peak twist may be increased and recoil rate unchanged, leading to reduced recoil rate relative to peak twist [91]. As the disease progresses, systolic dysfunction may lead to impaired recoil. The relationship between recoil rate and pressure drop in the LV also needs further investigation, correcting for the effects of peak torsion and end-systolic volume [43].

Population-based statistical models of cardiac function are now becoming available [95], in which regional wall motion can be mapped to a standard atlas of the heart and statistical tests performed to quantify the degree of abnormality. These atlases will be useful in characterizing the expected range of torsion in patient subgroups.

A combination of population imaging and mechanical modelling is required to understand the mechanism for the increased torsion found in the subendocardium, and the coupling mechanism which generates transverse shear and wall thickening. In particular, further work is needed on the torsion to shortening ratio to determine if the predictions of computational physiological models of cardiac mechanics [96] are experimentally verified. Higher resolution non-invasive strain imaging methods such as 3D transmural displacement imaging with DENSE MRI [86] show a lot of promise in this regard.

## Conclusions

Torsion is an important index of cardiac function and provides additional information on myocardial performance over and above standard pump function indices. Although it is readily performed as part of any CMR examination, standardized methods of calculation are recommended. Torsion provides information on the relative mechanical influence of subendocardial vs subepicardial fibers, and will be particularly useful in characterizing mechanisms through the customization of mathematical models to individual patient torsion.

## Competing interests

AAY and BRC act as consultants for Siemens Medical Solutions.

## Authors’ contributions

AAY performed the literature review. Both authors (AAY and BRC) contributed in the design and writing of the manuscript, and approved the final manuscript.

## Supplementary Material

Additional file 1 JCMR Torsion Movie Base.avi. Click here for file

Additional file 2 JCMR Torsion Movie Apex.avi. Click here for file
